# A rare variant of oromandibular limb hypogenesis syndrome: a case report of glossopalatal ankylosis

**DOI:** 10.3389/fdmed.2026.1776248

**Published:** 2026-05-01

**Authors:** Sumit Chopra, Vidya B, Ekta Singh, Ankit Parashar, Mahendra Dandge

**Affiliations:** 1 SRD Dental Clinic and Maxillofacial Surgery Centre, Dehradun, India; 2 Nitte (Deemed to be University), AB Shetty Memorial Institute of Dental Sciences (ABSMIDS), Department of Oral and Maxillofacial Surgery, Mangalore, India; 3 Private Practitioner, Dehradun, Uttarakhand, India; 4 Altrus HealthCare Hospital, Dehradun, India

**Keywords:** cleft palate, glossopalatine ankylosis, hypomelia, oromandibular limb hypogenesis syndrome, oromandibular limb hypoplasia

## Abstract

**Introduction:**

Oromandibular limb hypogenesis syndrome (OLHS) Type IIIA is an exceptionally rare congenital anomaly, with only a few cases documented in the literature. It is characterized by developmental hypoplasia of the tongue, mandible, and limbs, manifesting as hypoglossia, micrognathia, and hypomelia.

**Case presentation:**

We report the case of a 9-year-old patient presenting with restricted mouth opening and feeding difficulties. Clinical examination revealed fusion of the tongue to the palate with synechia. Surgical intervention involved the release of the dense fibrous band between the tongue and palate, followed by palatoplasty to correct the cleft palate. The cleft palate was successfully repaired using the Von Langenbeck technique, restoring oral functionality.

**Conclusion:**

This case highlights the clinical manifestations, differential diagnosis, and surgical management of OLHS Type IIIA. It underscores the importance of timely intervention to improve functional outcomes in patients with this rare syndrome.

## Introduction

Oromandibular limb hypogenesis syndrome (OLHS) is an extremely rare condition that mainly impacts the tongue, palate, and limbs, presenting with a range of overlapping clinical characteristics. These congenital anomalies affect the orofacial area, where notable deformities of the tongue and limbs are seen alongside developmental issues of the maxilla and mandible (1). Hall categorized OLHS into five types (Types I–V) based on oral and limb defects (2). Type IIIA, a variant of OLHS, is distinguished by an intraoral band of varying thickness that connects the tongue to the hard palate or the maxillary alveolar ridge. This syndrome has been linked to several associated abnormalities, including a cleft palate (in some instances, the tongue was occasionally attached to the nasal septum), mandibular hypoplasia, and upper-lip hypoplasia. Additionally, clinical manifestations include hypodontia and various limb anomalies, such as oligodactyly, syndactyly, and polydactyly (3, 4). The diagnosis of this syndrome is determined through a clinical triad that includes hypoglossia, micrognathia, and limb anomalies of varying severity (5). The estimated prevalence is approximately 1 in 100,000 live births, categorizing it as an extremely rare condition according to Orphanet. Glossopalatine ankylosis has been assigned the ORPHA code ORPHA:141163 (6). The cause of this condition remains uncertain, with both environmental and genetic factors proposed as potential contributors; however, environmental influences are thought to be more common (7, 8).

The other clinical manifestations include a receded chin, atrophic mandibular alveolar ridge, and missing incisors. Additional extraoral findings comprise a defective lower lip, hypertrophic major salivary glands, a midline mandibular cleft, an enlarged sublingual ridge, fibrotic bands connecting the lower lip to the alveolar ridge, and gingival abnormalities (9). This condition has been linked to functional impairments such as difficulties in oral feeding, a compromised airway, and restricted speech development, all of which present significant management challenges. Given the limited number of reported cases, each new report contributes to a deeper understanding of its clinical features, associated anomalies, and management strategies. In this report, we detail a case of glossopalatine ankylosis associated with a cleft palate and limb hypoplasia, discussing the complexities faced in its clinical management and emphasizing the necessity of a multidisciplinary approach that includes maxillofacial surgeons, pediatricians, anesthesiologists, and speech therapists.

## Case report

### Case presentation

A 9-year-old boy from Saharanpur, Uttar Pradesh, India, was admitted to the hospital with a primary complaint of being unable to open his mouth. He also experienced nasal regurgitation while feeding, difficulties with speech, and underdevelopment of all four limbs. He was the first child of a healthy 25-year-old mother and a 29-year-old father, with no history of consanguineous marriage. His parents, along with his two younger siblings and extended family, were all healthy and had no known history of craniofacial or skeletal abnormalities. The mother mentioned that she had taken an unknown medication for headaches, which she acquired from a pharmacy without a prescription during her first trimester of pregnancy. An antenatal ultrasound scan conducted at 22 weeks of gestation showed no abnormalities. The child was born at 36 weeks of gestation in a local maternity hospital via normal vaginal delivery, weighing 2.45 kg at birth. Upon birth, the infant displayed an inability to open his mouth, feeding difficulties, and limb malformations, leading to a referral to a pediatrician. The pediatric assessment indicated synechia due to glossopalatine ankylosis, along with a cleft palate and hypomelia affecting all four limbs. The infant was fed through a feeding tube starting from the second day of life until he turned 3 years old, with the Ryle's tube being replaced monthly. After reaching 3 years of age, he began feeding with a spoon.

**Figure 1:**
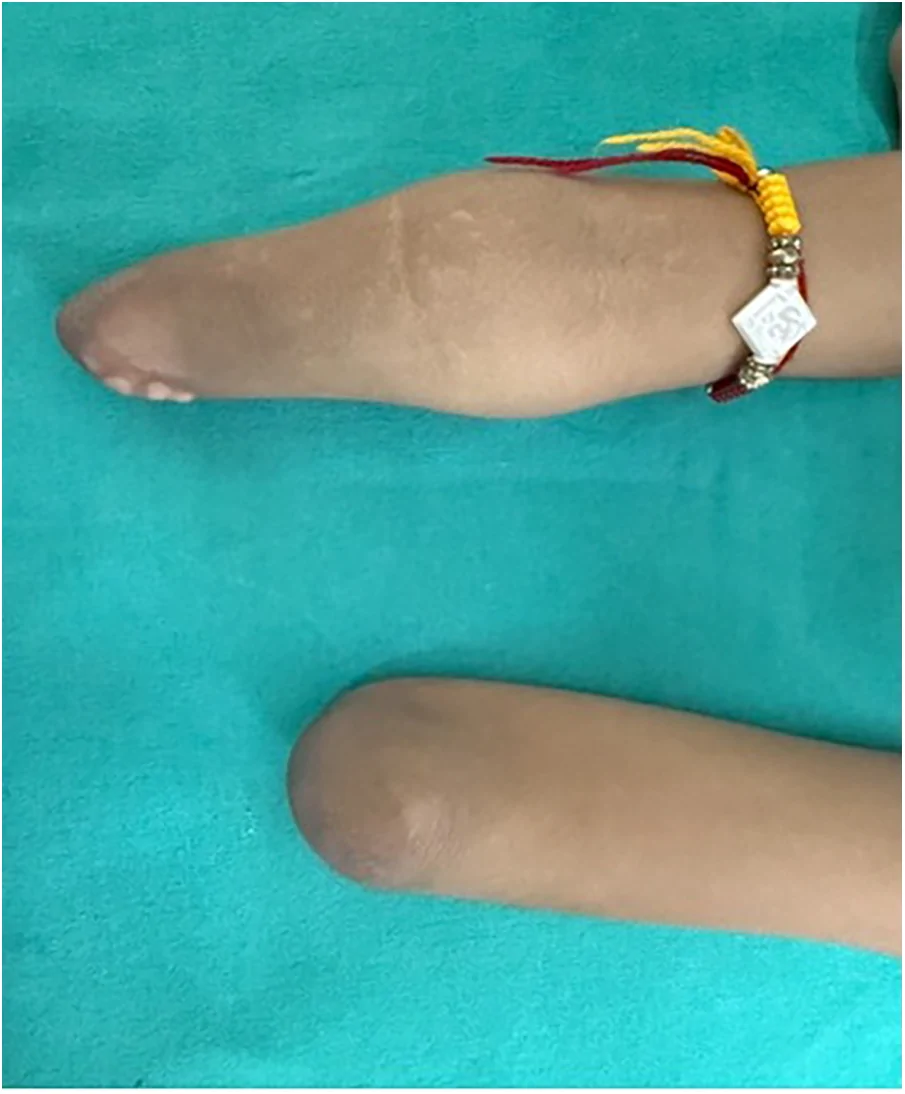
Two feet resting on a teal cloth, with both feet showing partial absence of toes and presenting rounded stumps; one ankle features a yellow and red thread bracelet with a silver ornament.

**Figure 2:**
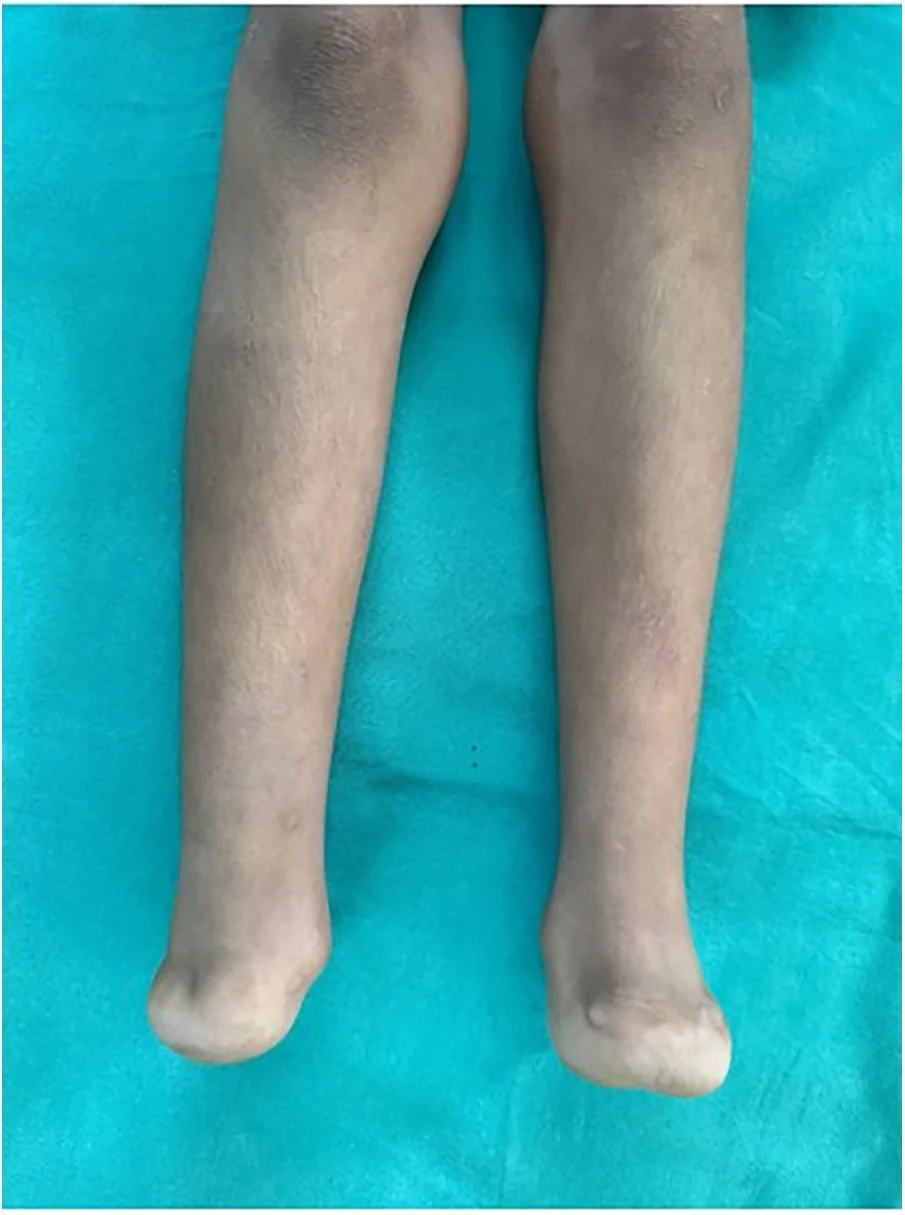
Lower legs of a person with both feet showing significant congenital deformities, characterized by the absence of properly formed toes and feet, displayed on a teal medical drape background.

**Figure 3:**
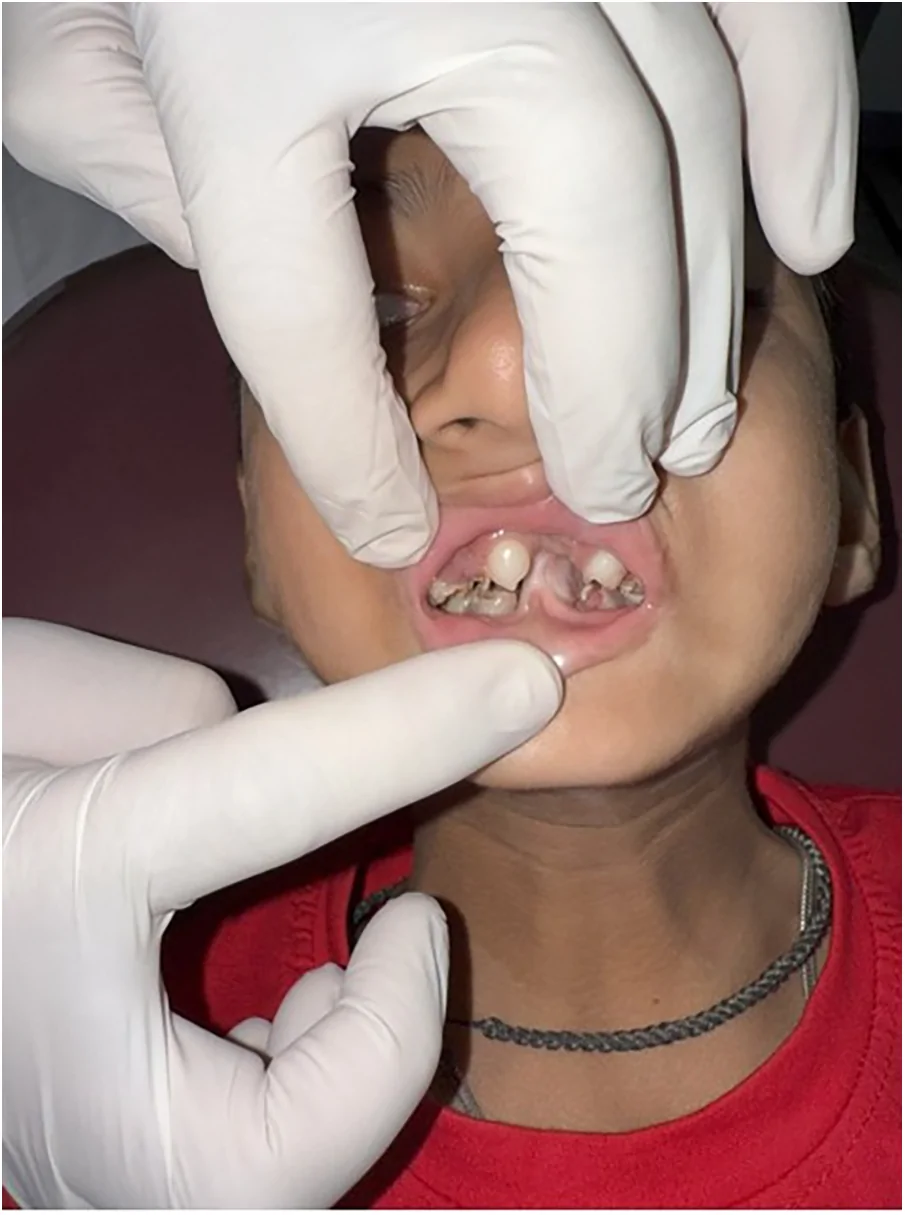
A dental professional wearing white gloves examines a child's upper front teeth, showing severely decayed teeth and a prominent dental abscess or swelling in the upper gum area.

At the age of 3, the patient had surgery to release adhesions, which initially improved mouth opening; however, the condition recurred within a year. When the patient presented to our hospital at 9 years old, he had limited mouth opening and significant speech difficulties, with unclear articulation that was only understandable to his parents. There was no reported history of hearing issues or learning disabilities, and no other systemic disorders were found. An extraoral examination showed facial hypoplasia, including a hypoplastic maxilla and mandible, along with retrognathia. Limb anomalies were also observed, manifesting as micromelia in all four limbs. The upper limbs exhibited varying degrees of distal hypomelia, with a complete absence of carpal, metacarpal, and digital bones (Figure 1). The left arm had a well-formed upper arm, but the forearm was missing. The right upper limb displayed a well-formed arm and forearm, yet the hand, palm, and fingers were absent. Both lower limbs showed apodia, with the absence of tarsal, metatarsal, and phalangeal bones (Figure 2). An intraoral examination indicated fusion of the palate with the dorsum of the tongue, leading to a complete lack of mouth opening (Figure 3). The tongue was hypoplastic, and the mucosa of the floor of the mouth was continuous with the palate. A cleft palate and a shallow vestibule were also noted, with the upper and lower alveolar ridges closely approximated. Salivary flow appeared to be normal. A total of 11 teeth were present in the oral cavity, comprising both deciduous and permanent dentition. The deciduous teeth present were 54, 55, 64, 65, 75, and 85, while the permanent teeth identified were 13, 16, 26, 36, and 46 (Table 1).

**Table 1:** Table 1 showing the dentition mentioned according to FDI system seen in the patient.

Teeth seen inside the oral cavity of the patient represented in FDI system							
Maxillary teeth	16	55	54	13	64	65	26
Mandibular teeth	46	85				75	36

Based on these clinical findings, we arrived at the diagnosis of oromandibular limb hypogenesis syndrome Type IIIA.

An orthopantomogram (OPG) indicated the presence of hypodontia, showing teeth 54, 55, 64, 65, 74, 75, 84, 85, 13, 16, 26, 36, and 46. Other bony structures, including the temporomandibular joint **(**TMJ) with normal joint space and the maxillary sinuses, appeared to be normal. A prominent antegonial notch was observed on both sides. An irregular or wavy outline of the inferior border in the anterior region of the mandible on an OPG typically suggests a technical error during imaging, often due to patient movement. The radiographic images of the affected limbs were not accessible. The management plan for this patient involved a multidisciplinary approach. The initial treatment focused on surgically relieving the glossopalatine ankylosis to restore function. Informed consent was obtained in writing, and the surgical procedure was performed under general anesthesia. Given the patient's preexisting limited mouth opening, a tracheostomy was carried out to ensure airway security during the surgery. Local anesthesia with lignocaine 2% and 1:200,000 adrenaline was used to infiltrate the maxillary anterior area. An incision was made with a No. 15 BP blade, extending in an anteroposterior direction.

**Figure 4:**
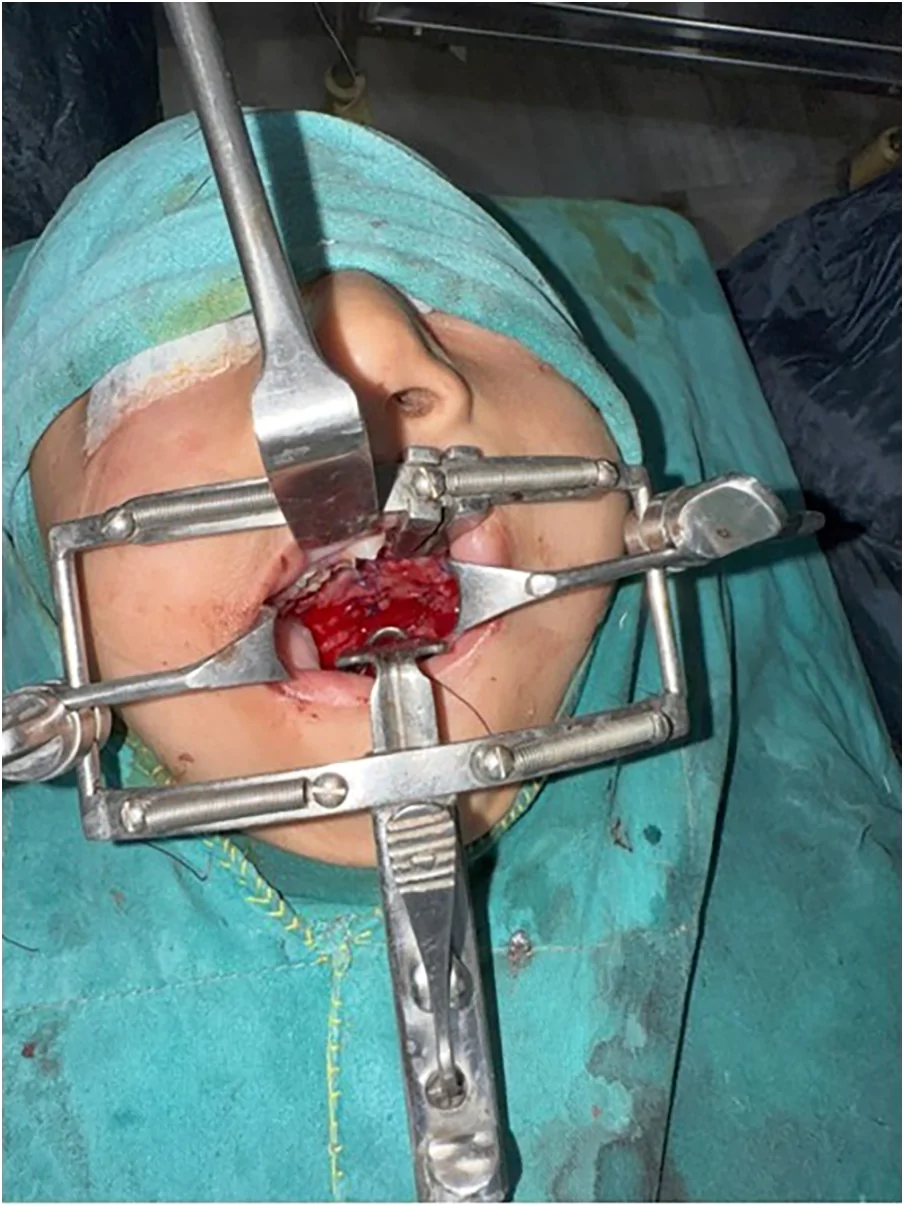
Surgical scene showing a patient under anesthesia with mouth held open by a metal retractor, exposing oral tissues and surgical instruments in use, indicating an ongoing oral or maxillofacial procedure.

**Figure 5:**
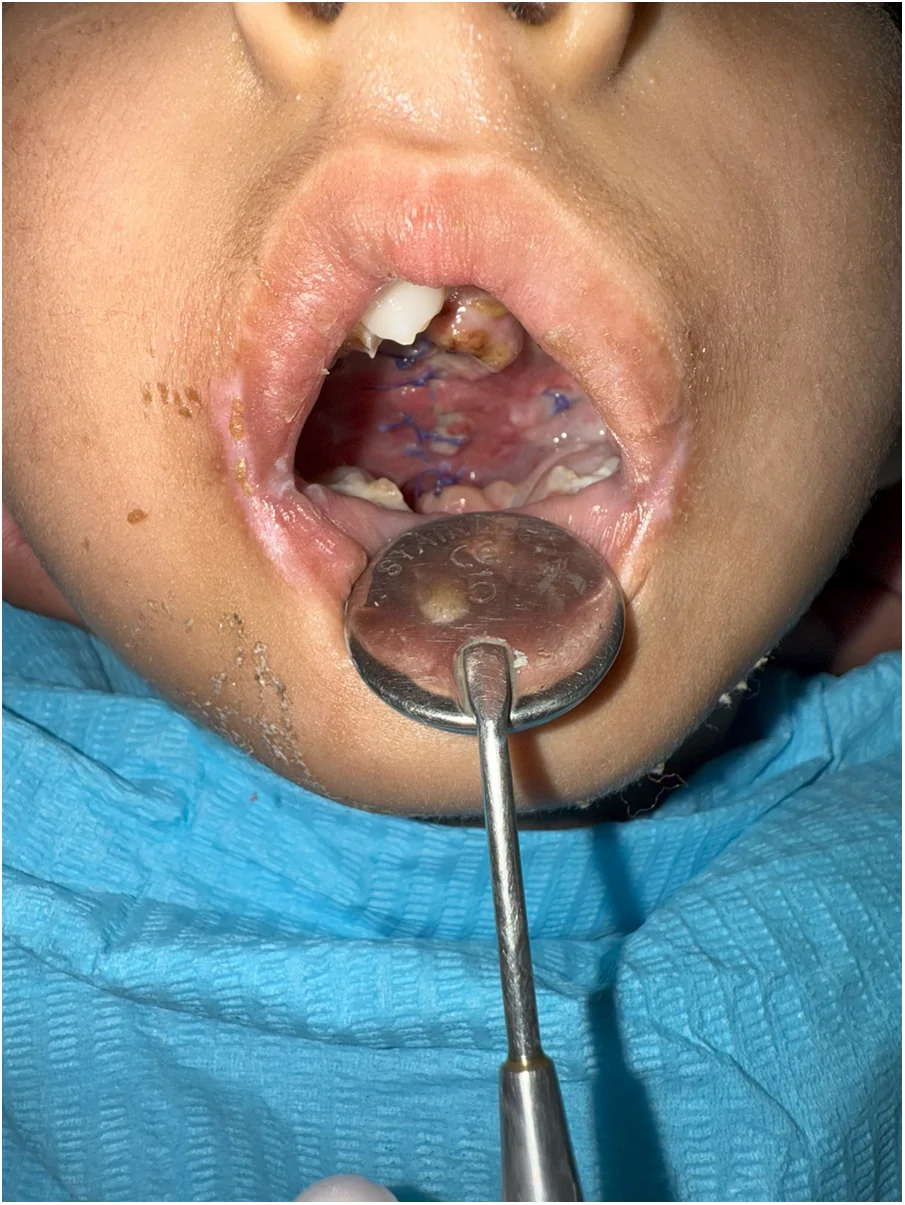
Close-up view of a child's open mouth during a dental examination, showing exposed upper gums with visible surgical sutures and healing tissue, a broken upper front tooth, and a dental mirror reflecting the oral cavity.

**Figure 6:**
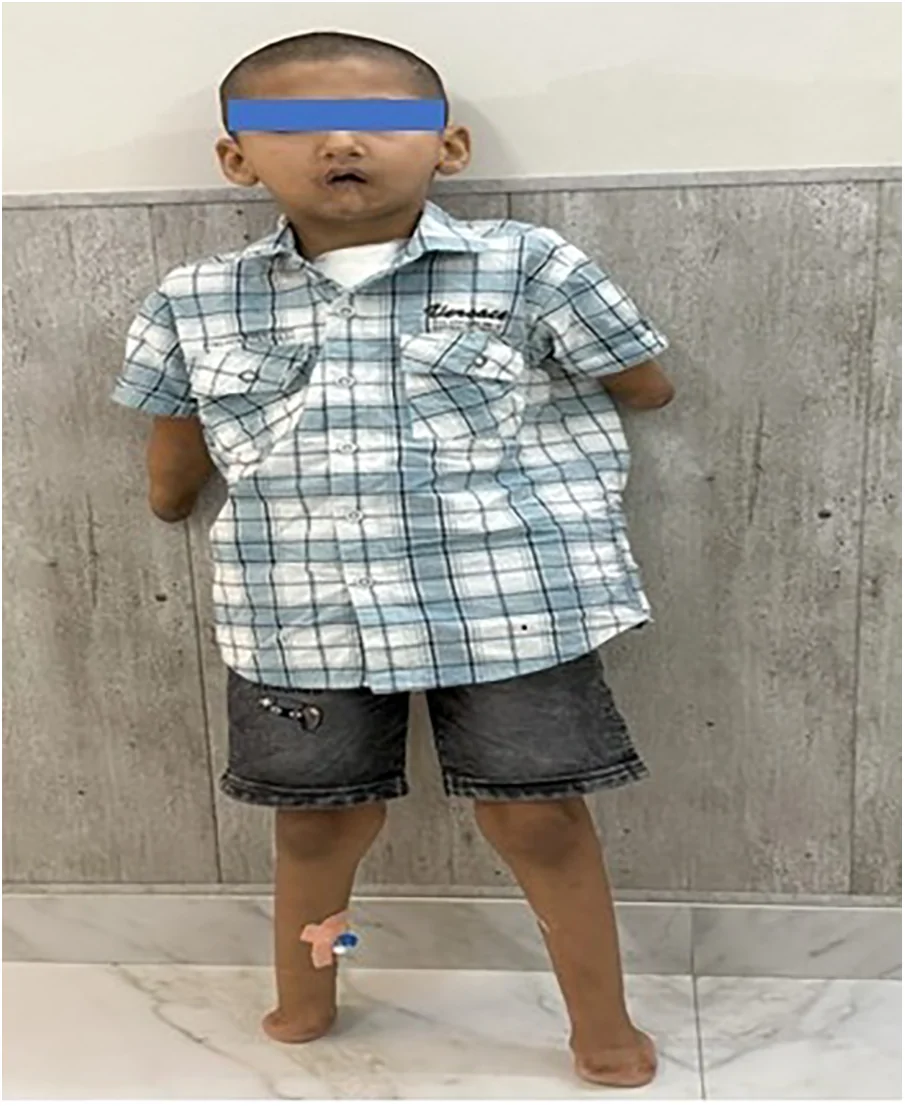
Clinical photograph depicting hypoplasia involving all four limbs.

The incision was made on the mucosa and fibrotic band that connected the anterior palate to the front part of the dorsal surface of the tongue, extending to the back section at the junction of the normal palatal mucosa and the palatal cleft mucosa. The mucosal and fibrotic bands were entirely separated, and hemostasis was achieved through cautery. A Heister mouth gag was then employed to maintain the surgical field, achieving a mouth opening of 35 mm. Subsequently, palatoplasty was performed using the Von Langenbeck technique, a recognized surgical approach for cleft palate repair (Figure 4). Incisions were made along the medial edges of the cleft, with releasing incisions created posterior to the maxillary tuberosity and along the alveolar process. Blunt dissection was conducted between the superior constrictor muscle and the velar musculature. The nasal mucosa was approximated with simple interrupted sutures using 4-0 Vicryl suture material. The uvula was closed with horizontal mattress sutures, followed by a layered closure of the soft palate (Figure 5). The adhesions between the tongue and the floor of the mouth were released using a No. 15 BP blade. After dissection, the raw surfaces on the ventral side of the tongue and the floor of the mouth were covered with a collagen sheet. The patient was placed on regular follow-up to monitor his functional recovery and was referred to a speech therapist for speech rehabilitation. At the 6-month follow-up, the patient had made a good recovery without complications or recurrence of synechiae, and an improvement in speech was observed (Figure 6).

## Discussion

Aglossia and hypoglossia are developmental disorders affecting the tongue, with the first documented occurrence of aglossia and adactylia noted by Rosenthal in 1932 (10). In 1971, Hall identified a link between hypoglossia and limb anomalies, coining the term oromandibular limb hypogenesis. He categorized this syndrome into five main types, with hypoglossia as a key criterion. Hall's classification of OLHS remains the most widely accepted to this day (Table 2). Consequently, the current case was clinically identified as oromandibular limb hypogenesis Type IIIA, characterized by hypoglossia, glossopalatine ankylosis, mandibular micrognathia, and hemimelia. This condition is exceedingly rare, with only a few cases documented thus far (11). Chicarilli and Polayes proposed a classification of OLHS based on embryological origins and clinical characteristics, where hypoglossia served as the primary factor for categorization (12). In 1981, Lustmann et al. recommended that the following three critical features be present for the diagnosis of OLHS: hypoglossia, hypomelia, and micrognathia (5).

**Table 2:** Hall's classification (1971) of oromandibular limb hypogenesis syndrome.

Type I	Salient features
Type IA	Hypoglossia
Type IB	Aglossia
Type II	
Type IIA	Hypoglossia-hypodactylia
Type IIB	Hypoglossia-hypomelia
Type IIC	Hypoglossia-hypodactylomelia
Type III	
Type IIIA	Glossopalatine ankylosis (ankylossum superius syndrome)
Type IIIB	With hypoglossia
Type IIIC	With hypoglossia-hypodactylia
Type IIID	With hypoglossia-hypomelia
Type IIIE	With hypoglossia-hypodactylomelia
Type IV	
Type IVA	Intraoral bands and fusion
Type IVB	With hypoglossia
Type IVC	With hypoglossia-hypodactylia
Type IVD	With hypoglossia-hypomelia
Type IVE	With hypoglossia-hypodactylomelia
Type V	
Type VA	Hanhart syndrome
Type VB	Charlie M syndrome
Type VC	Pierre Robin Syndrome
Type VD	Mobius syndrome
Type VE	Amniotic band syndrome

The cause of OLHS is still not fully understood; nonetheless, both environmental and genetic factors are believed to significantly contribute. Environmental influences such as maternal hyperthermia, intrauterine trauma, exposure to radiation, medications, and nutritional deficiencies during pregnancy are thought to be linked to OLHS. Additionally, teratogenic agents have been identified as a significant cause of glossopalatine ankylosis syndrome (13). Numerous case reports have indicated that maternal exposure to substances such as trimethobenzamide hydrochloride, meclizine hydrochloride, and marijuana during pregnancy may serve as a cause; however, definitive evidence connecting these exposures to negative fetal outcomes has yet to be established. In one instance, the mother was also noted to have been exposed to radiation in the first trimester (3). Infection with SARS-CoV-2 during the first trimester could lead to the development of hypoglossia and micrognathia, likely occurring together due to an unknown factor that may impact the first branchial arch during organogenesis (14). The *MSX2* homeobox gene, found in the mesenchyme of branchial arches, developing teeth, and limb buds, plays a crucial role in the formation of craniofacial structures, limbs, and body axes. A pathogenic variant of *MSX2* may be a genetic cause of OMLH, although this has not been definitively proven (15). This aligns with the organs affected in OMLH, which leads to the observed clinical symptoms. However, a distinct genetic factor has not been associated with OMLH, and the majority of cases appear to be sporadic. In our case, the patient did not receive an evaluation from a clinical genetics service, and no molecular studies were conducted to investigate the pathogenic variant.

The formation of the tongue, palate, and limbs occurs within a close timeframe, specifically between the 4th and 8th weeks of intrauterine development. During the process of organogenesis, the emergence of vascular defects could potentially explain the malformations observed in the distal areas of the tongue, palate, and limbs (16). Hypoplasia of the tongue can vary significantly, ranging from complete absence (aglossia) to minimal hypoplasia, which may also involve a hypoplastic mandible and a midline cleft. It has been proposed that damage or premature involution of the stapedial artery, which supplies the first branchial arch, could disrupt the vascularization of the tongue (17). The most diverse findings are associated with limb development and range from syndactyly to the complete absence of limbs; thus, variations are consistently found distal to the humerus and femur. Involvement may affect a single limb or multiple limbs. Additionally, visceral anomalies may present as conditions such as renal agenesis, an imperforate anus, a fused labia majora, and ileal atresia (5).

In this particular case, the patient's mother took medication for headaches during the first trimester of her pregnancy. We hypothesize that the fetus may have experienced some impairment or injury due to the unidentified drug that was consumed. It could also represent a sporadic occurrence, as only one individual in the family has been affected, as indicated by Meundi et al. in 2013 (17). Ergotamine, a vasoconstrictive medication used for headache relief, is considered a possible teratogenic agent in OMLH. This drug causes vasoconstriction in the human cerebral vasculature, resulting in ischemia and tissue necrosis, which can lead to malformations. Caffeine, acting as a vasodilator, works in conjunction with ergotamine, exacerbating the vasoconstriction in the cerebral vessels (18). Makhoul et al. in 2003, through their epidemiological research, proposed that congenital limb deficiency is a relatively common diagnosis among live-born infants. Detailed prenatal fetal ultrasonography can enable early detection, providing essential information to parents and helping them make informed decisions regarding the future of the pregnancy (19).

The primary clinical issue that requires urgent attention is synechia, as it is linked to difficulties in feeding, which can lead to weight problems and hinder the normal growth of newborns. In this particular case, the patient was reliant on a feeding tube from the second day of life until the age of 3, after which feeding was transitioned to a spoon. The synechia was addressed surgically, with the adhesions being excised and palatoplasty performed. A significant challenge in managing this case was the lack of tissue in the hypoplastic tongue and cleft palate (20). Typically, a palatal defect necessitates adequate tissue for a tension-free closure to avoid the development of an oroantral fistula during the postoperative phase. Moreover, excessive incision of the soft tissue from the dorsal side of the tongue may lead to impaired speech. Effectively managing such patients is complex, and a multidisciplinary approach is recommended for optimal treatment outcomes (21). This should include surgical procedures, orthodontic care, and preventive strategies such as oral prophylaxis, pit and fissure sealants, and consistent fluoride therapy. The degree of oral synechia is typically assessed through physical examination alone, which is adequate in the majority of instances; however, it must be distinguished from other similar conditions (22). OMLH Type IIIA can be differentiated from other syndromes that exhibit hypoglossia or limb deformities (Table 3). These include Moebius syndrome, Hanhart syndrome, Charlie M Syndrome, Pierre Robin syndrome, amniotic band syndrome, splenogonadal fusion, and otocephaly (17, 22). To prevent the recurrence of tongue and palate fusion following surgery, physical barriers such as tissue-based flaps, splints, and fibrin glue are typically employed to maintain separation of the surfaces, along with careful implementation of postoperative exercises (23–26).

**Table 3:** Syndromes presenting with hypoglossia and limb anomalies.

Syndrome	Inheritance	Orofacial	Skeletal	Other
Charlie M	Sporadic, prevalence unknown (<5 cr.)	Facial asymmetry, hypertelorism, telecanthus, short philtrum, micrognathia, microstomia, aglossia/hypoglossia, absent teeth, cleft palate, gingival fibromatosis, glossopalatine ankylosis	Ectromelia, ectrodactyly, oligodactyly	
Hanhart	Sporadic; autosomal dominant <1:1,000,000	Facial asymmetry, hypertelorism, telecanthus, short philtrum, micrognathia, microstomia, aglossia\hypoglossia, absent teeth, cleft lip/palate, glossolabial adhesion	Ectromelia (all limbs), ectrodactyly, oligodactyly	Cases of gastroschisis and pulmonary hypoplasia
Möbius	Sporadic, autosomal dominant, prevalence unknown	Clinical features of Hanhart; CN 7 defect (major criteria); CN III, IV, VI, IX, X, and XII defects possible	Clinical features of Hanhart	Clinical features of Hanhart
Pierre Robin	Sporadic, autosomal dominant or autosomal recessive associated with 17q23, SOX9	Cleft palate, micrognathia, glossoptosis	Clinodactyly	20–40% isolated, otherwise part of other syndromes such as Stickler or campomelic dysplasia

## Conclusion

This case report presents a very rare presentation of OMLH type IIIA and provides an insight into numerous proposed etiological factors and clinical presentation. It also provides a special emphasis on surgical approaches and challenges in the management and prevention of synechia recurrence.

## Data Availability

The original contributions presented in the study are included in the article/Supplementary Material. Further inquiries can be directed to the corresponding author.
